# Stakeholder engagement in methodological research: Development of a clinical decision support tool

**DOI:** 10.1017/cts.2019.443

**Published:** 2020-02-18

**Authors:** Denise H. Daudelin, Robin Ruthazer, Manlik Kwong, Rebecca C. Lorenzana, Daniel J. Hannon, David M. Kent, Timothy E. McAlindon, Norma Terrin, John B. Wong, Harry P. Selker

**Affiliations:** 1 Tufts Clinical and Translational Science Institute, Tufts University, Boston, MA, USA; 2 Institute for Clinical Research and Health Policy Studies, Tufts Medical Center, Boston, MA, USA; 3 School of Engineering, Tufts University, Medford, MA, USA; 4 Predictive Analytics and Comparative Effectiveness (PACE) Center, Institute for Clinical Research and Health Policy Studies (ICRHPS), Tufts Medical Center, Boston, MA, USA; 5 Division of Rheumatology, Tufts Medical Center, Boston, MA, USA; 6 Division of Clinical Decision Making, Tufts Medical Center, Boston, MA, USA

**Keywords:** Stakeholder engagement, shared decision-making, decision support, knee osteoarthritis, total knee replacement, predictive models

## Abstract

**Introduction:**

Shared patient–clinician decision-making is central to choosing between medical treatments. Decision support tools can have an important role to play in these decisions. We developed a decision support tool for deciding between nonsurgical treatment and surgical total knee replacement for patients with severe knee osteoarthritis. The tool aims to provide likely outcomes of alternative treatments based on predictive models using patient-specific characteristics. To make those models relevant to patients with knee osteoarthritis and their clinicians, we involved patients, family members, patient advocates, clinicians, and researchers as stakeholders in creating the models.

**Methods:**

Stakeholders were recruited through local arthritis research, advocacy, and clinical organizations. After being provided with brief methodological education sessions, stakeholder views were solicited through quarterly patient or clinician stakeholder panel meetings and incorporated into all aspects of the project.

**Results:**

Participating in each aspect of the research from determining the outcomes of interest to providing input on the design of the user interface displaying outcome predications, 86% (12/14) of stakeholders remained engaged throughout the project. Stakeholder engagement ensured that the prediction models that form the basis of the Knee Osteoarthritis Mathematical Equipoise Tool and its user interface were relevant for patient–clinician shared decision-making.

**Conclusions:**

Methodological research has the opportunity to benefit from stakeholder engagement by ensuring that the perspectives of those most impacted by the results are involved in study design and conduct. While additional planning and investments in maintaining stakeholder knowledge and trust may be needed, they are offset by the valuable insights gained.

## Introduction

When incorporated into shared patient–clinician decision-making, decision support tools or decision aids have been found to improve patient knowledge and to help patients clarify their personal values and make value congruent decisions with their clinician [1,2]. In severe osteoarthritis, shared patient–clinician decision-making is central to choosing between nonsurgical treatments and surgical knee replacement (TKR) as a preference-sensitive care choice. Although the harms and benefits of the options in decision support tools are typically based on aggregate data, in this project we set out to develop a decision support tool that would be targeted to individual-specific characteristics by using predictive models. To make those models relevant to patients with knee osteoarthritis and their clinicians, as stakeholders, we involved patients, family members, patient advocates, specialist, generalist, and allied health clinicians, and researchers in all aspects of the project.

The incorporation of decision support in elective surgery, in general, and for knee osteoarthritis treatment in particular, is intended to improve patients’ knowledge of treatment options and provide balanced information on clinical outcome probabilities. Research from clinical trials suggests that many elective surgical procedures are performed in the context of patients’ inflated perceptions of the probabilities of benefits with lack of appreciation of the risk for harms and of awareness of treatment alternatives [3-6]. The implementation of patient decision aids for some preference-sensitive health conditions may both reduce the rates of elective surgery and lower costs [2,7].

The engagement of stakeholders in research is still evolving in its terminology and frameworks [8,9]. The stakeholder definition we used for this project, intended for comparative effectiveness research (CER), was “individuals, organizations, or communities that have a direct interest in the process and outcomes of a project, research, or policy endeavor [9].” There are variations in the expectations accompanying stakeholder engagement. Researchers have defined this as a repetitive process that seeks the “knowledge, experience, and judgment and values of individuals selected to represent a broad range of direct interests in a particular issue, for the dual purposes of creating a shared understanding and making relevant, transparent and effective decisions [9].”

Stakeholder engagement in community-based participatory research and participatory action research, and increasingly in CER, has been well documented and represented in the literature [10,11]. Stakeholder engagement is less common in research that involves evidence synthesis, integration, dissemination, and application [12], and there is little literature for stakeholder engagement in methods research such as the development of predictive models for patient-specific shared decision-making that we describe here.

In this project, we developed the Knee Osteoarthritis Mathematical Equipoise Tool (KOMET) to provide patient-specific decision support for shared decision-making when choosing between nonsurgical treatments and surgical TKR. We incorporated stakeholder perspectives to ensure that the predictive models and user interface were relevant to patient–clinician decision-making. This paper describes the process and outcomes of this engagement.

## Methods

We engaged stakeholders in all aspects of the project. The development of the models and the proposed use of KOMET have been described previously [13]. In summary, to develop KOMET’s predictive models for clinical outcomes of TKR and of nonsurgical treatments, we created a consolidated database with treatment outcomes of knee severe osteoarthritis from a variety of clinical study and registry data. Model variables were selected based on the input from patients and clinicians and on variables’ contributions to models’ predictive performance. These models were then incorporated into decision support software prototypes that were pilot tested with stakeholders, clinicians, and patients.

Stakeholders were selected to represent the range of individuals or groups responsible for, or affected by, health- and healthcare-related decisions about knee osteoarthritis (OA) treatments [14]. The stakeholder engagement plan was shaped by the Patient-Centered Outcomes Research Institute (PCORI) engagement principles [15], and stakeholders were involved in all aspects of the project including:

- selection of *study questions and outcomes*,
- selection of candidate variables for *creating the modeling database and predictive models*, and
- *user interface development* and testing.

### Selection of Stakeholders and Engagement Activities

Two stakeholder panels, one of patients, families, and patient advocates and another of a range of clinicians and researchers, were created to structure and facilitate engagement. The seven patient stakeholder panel members (four women and three men) were identified through discussions with clinicians, knee osteoarthritis researchers, and the Arthritis Foundation. The seven clinician and researcher panel stakeholders (three women and four men) were recruited among local primary care, orthopedic, and rheumatology clinicians and researchers. The Principal Investigator, Project Director, Research Assistants, Statistician, and other research team members participated in the panels by facilitating discussions and providing education on study methods and topics important to stakeholders. Stakeholder panel in-person group meetings were held at least quarterly throughout the project with additional conference calls and one-on-one meetings used to gather in-depth feedback from members or test the decision support user interface.

Our engagement activities were designed to address PCORI engagement principles of reciprocal relationships, partnerships, colearning, and transparency, honesty, and trust [15,16].

Reciprocal relationships were supported by engaging stakeholders as research team member participants in regularly scheduled meetings or conference calls and using their guidance for decision-making throughout the study. We engaged all stakeholders as project partners with consideration given to the effective use of their time, their occasional need to limit project participation due to acute care needs, professional responsibilities, and other obligations. Patient and family stakeholder meetings were 90 minutes long and scheduled to avoid peak travel times. Clinician meetings were held as working lunches to accommodate clinic schedules. Equal compensation was provided for patients, families, and clinicians.

The colearning principle was addressed by ensuring that stakeholders gained the needed knowledge about predictive modeling to contribute their perspectives to the model and user interface development process. Patient stakeholder training on decision support model development included a presentation about the modeling process using common examples such as predicting a person’s weight based on their age and gender. Clinician training included a discussion of decision support research and decision support examples such as using statin medications for preventing cardiovascular events. Patient and clinician panel meetings were conducted separately so that the content and terminology addressed the interests and learning needs of the members. We facilitated discussions to ensure participation of all stakeholders, providing clarifications as needed and encouraging stakeholder questions. Also, panel meetings fostered honest discussions about what mattered most to patients and clinicians involved in the shared decision-making process and how it should shape the design and dissemination of the decision support tool.

The last principle, which includes transparency, honesty, and trust, was evident in the open communication fostered throughout the project. The intent was to share with stakeholders the successes and challenges of the project and to learn from stakeholders if the project’s direction and resulting decision support tool were addressing stakeholder needs.

### Selection of Study Questions and Outcomes

Stakeholder engagement began during the planning of the research project with focused stakeholder discussions about potential study questions. Specifically, we asked stakeholders to consider “If an adult with knee osteoarthritis is presented with medical and surgical treatment options, what decision support could be provided at the point of care that the clinician and patient can use to understand the patient-specific predicted outcomes of importance to the patient?” During these discussions, patients and clinicians described the importance of knee osteoarthritis treatments that alleviate pain and return people to their desired level of physical functioning.

In the project’s initial stages, we confirmed and clarified the selection of knee pain and overall function as targets for the development of predictive models and decision support. Stakeholder panel meetings included facilitated discussion of patient and clinician experiences in making knee osteoarthritis treatment decisions and pain- and function-related factors that influenced these preference-sensitive decisions. These conversations also included other treatment considerations such as risk factors, family support, rehabilitation, employment and retirement considerations, and socioeconomic issues. Patient and clinician panels were also asked how factors such as the joint replacement lifespan and the impact of multiple comorbidities affected considerations about the timing and desirability of knee replacement. Patients who already had TKR and clinicians who treat such patients were asked about the timeframe at which the benefits of the TKR were realized in order to select a time point for predicted outcomes. The responses to these questions informed study outcome selection, the creation of the study database, and the development of the predictive models.

### Stakeholder Engagement in Creating Modeling Database and Developing Predictive Models

The database created for development of the predictive models for pain and function combined four datasets, and stakeholder input influenced decisions about which of the many potential data elements would remain in the database. A variable ranking tool using a rating scale was used to gather stakeholder opinions about the importance (not at all important to very important) and ease of collection (very easy to very hard) of each variable in predicting the degree of pain relief and improved function that someone would receive from knee replacement. Variable ranking was summarized by importance and ease of collection. A fifth dataset was acquired but not used since it did not include adequate data for the ultimately adopted study design, predictors, and outcomes.

Because the knee pain and overall function predictive models would be based on patient-reported pain and function scales, we sought patient and clinician feedback on the meaningfulness of the various assessment questions. We also sought input on topics not addressed by these tools. Table 1 provides examples of stakeholder discussion questions. Also, because the modeling database would be composed of matched patients with and without TKR, to allow comparisons of their outcomes, we sought guidance from clinicians, researchers, and patient stakeholders and used results from prior published literature [17] to determine key variables for creating these matched pairs.

**Table 1.** Examples of stakeholder discussion questions to solicit the feedback needed for creating the modeling database and developing predictive models

**Patient, family and advocate discussion questions:**
How meaningful are the pain scale questions to you? Are there important aspects of pain that aren’t included in these questions? How meaningful are the health survey (functional assessment scale) questions to you? Are there important aspects of function that aren’t included in these questions? What future period would you want to consider as you make a treatment decision? What is your pain and function in 3 months, 6 months, 1 year or 2 years? How will you take into account the rehabilitation period after total knee replacement when making a decision between treatments?
**Clinician and researcher discussion questions:**
What has your experience been like in making knee osteoarthritis treatment decisions? What has your experience been like in working with patients making knee osteoarthritis treatment decisions? What variables do you think are most important to include in a predicative model of knee osteoarthritis outcomes?

Patient and clinician panels helped select predictive model variables, and after model development, they provided input on the clinical importance of the models’ results. Based on the initial development of regression models, we provided a selection of variables that possibly could interact with the treatment effect including age, gender, knee pain, comorbidities, physical and mental function and led discussions with the patient and clinician panels about variable usefulness. Interaction variables allow the models to predict different benefits of TKR for different patients, so getting input on plausible interactions was important. We asked clinician stakeholders to rank the candidate main effect and interaction variables in order to include in the model selection process those considered important, plausible, and easily and reliably provided by patients. In order to select outcome variables, we asked stakeholders to rank how much the candidate variable would be related to pain and functional outcomes a year in the future (Table 2).

**Table 2.** Potential model variables clinicians ranked as fairly or very important to include in the model selection process

Gender Age Mental well-being Physical well-being Knee pain scale in problem knee Hip pain	Activities of daily living Narcotics Back pain Prior hip surgery Quality of life scale Bodily pain scale

### Stakeholder Engagement in User Interface Development and Testing

Throughout the project, we asked stakeholders how frequently, and at what point in the disease progression, they might expect to use the predicted outcomes in decision-making. We also discussed if the decision aid should be designed to be used at home before a clinical appointment or only in the office setting where they could receive support and guidance to interpret the predicted outcomes.

Lastly, we enlisted patient and clinician stakeholders to help design the KOMET user interface through which the patients would enter input to have their predicted outcomes displayed. In collaboration with a human factors engineer and user interface design experts, we developed a usability testing script and employed a “think aloud” protocol [18]. We provided user interface design prototypes to accomplish the following tasks: (1) orient the user to the purpose of the tool; (2) collect demographic, comorbidity, and model input variables; and (3) display the patient-specific model predictions for shared decision-making. During one-on-one meetings, stakeholders were asked (1) if the explanations about how to use the tool were clear and easy to understand, (2) if the questions and data collection formats were clear and easy to complete, and (3) if the predictive model displays were meaningful and would support decision-making. Stakeholder feedback led to revisions in all aspects of the tool. Repeat testing was conducted until stakeholders had no further suggestions for improvement.

The final version of the tool was tested with five clinicians on the stakeholder panel and a total of ten of their patients with knee osteoarthritis in the clinical setting to gather additional information about usability and usefulness for shared decision-making. Participating clinicians asked their patients who were considering surgical versus nonsurgical treatment options if they were interested in testing the tool and user interface. Patients completed the data collection portion of the program in an exam room prior to seeing the physician and were asked a series of questions by a study team member to determine if they could correctly describe the results of the decision support tool. The clinician then engaged the patient in a conversation about the KOMET results and the patient’s treatment goals and options.

## Results

### Stakeholder Engagement

The patient stakeholder panel included individuals (1) at risk for knee osteoarthritis due to osteoarthritis in other joints, (2) actively considering treatment options for their knee osteoarthritis, and (3) who previously had TKR for severe knee osteoarthritis. The clinician panel included two rheumatologists, two primary care physicians, two orthopedic surgeons, and one physical therapist. Both of the rheumatologists and one of the orthopedic surgeons were also researchers themselves and were able to represent the researcher perspective. Seventy-one percent (5/7) of the patient stakeholders and all of the clinician and researcher stakeholders remained engaged throughout the project, participating in each aspect of the research from determining the outcomes of interest to providing input on the design of the user interface displaying outcome predications. Two patient participants discontinued their participation in the last year of the study. Some patients, family members, and clinicians had to limit their participation due to personal or health issues and employment and clinical care responsibilities. About half of the patient stakeholders participated through conference calls due to travel, scheduling, and health considerations. Clinicians who could not attend group meetings were engaged individually to ensure their continued participation.

### Selection of Study Questions and Outcomes

Stakeholders strongly supported using both the knee pain and functional outcomes in patients’ decision-making processes. Our choice of comparators and outcomes, improvement in physical function and pain relief, reflects these as the two primary concerns of the patients and clinicians. The two continuous scale outcomes on which we built our models, the one-year knee pain from the Western Ontario and McMaster Universities Osteoarthritis Index (WOMAC) [19] and the 12-Item Short-Form Health Survey (SF-12) Physical component scores [20,21], were chosen after discussions with clinician and patient stakeholders. Other forms of outcomes considered included a percent change from baseline and dichotomizing these continuous measures into positive versus negative outcomes. We also considered factors such as the timeframe of the outcome assessment following recovery from surgery.

Discussions with stakeholders revealed that considerations of treatment outcomes were nuanced and that pain and function were deeply interconnected. Stakeholders and clinicians described the importance of pain relief in order to improve physical and emotional functioning. This led us to focus on both pain and function rather than selecting a single outcome. Stakeholders also discussed a range of other outcomes and considerations that play important roles in treatment decision-making that they believed should be included in a decision support tool, such as cost, rehabilitation considerations, lost time at work, future quality of life, and risks. These factors were beyond the scope of this iteration of KOMET due to limitations in the outcome variables available in the dataset. Future revisions of KOMET will attempt to address some of these additional outcomes important to stakeholders.

### Modeling Database and Predictive Model Development

Stakeholder engagement influenced the development of the modeling database and of the predictive model through their insights into the meaningfulness and interpretation of pain and function survey questions, the importance of postoperative factors in decision-making, and the ease of collection of variables. Physician and researcher stakeholders influenced the selection of the variables used for matching patients who had surgical treatment with ones who had nonsurgical treatment by identifying the available variables that would be clinically meaningful in defining a candidate for knee surgery.

Clinician and patient stakeholders provided input on the model’s variables, the clinical significance and feasibility of collecting the variables, and the results of the predictive models. The interaction terms in the regression models allow for individualized benefit predictions for different patients as related to the treatment options. Thus candidate primary and interaction variables included in the selection process were those considered important, plausible, and easily and reliably provided by stakeholders. Once the model was developed and the user interface was being designed, the research team and stakeholders decided that many of the variables under consideration were burdensome to collect or difficult to capture in a consistent manner. For example, the data collection required to compute a depression score initially included in the model was determined to be too burdensome. Thus, we removed this score as a variable from the model, confirmed that the performance characteristics (*r*-square, calibration) were not thereby unacceptably diminished, and moved forward with the revised model.

### Stakeholder Engagement in User Interface Development and Testing

Both the clinician and patient stakeholders contributed extensively to the design of the decision support application’s user interface and features. Stakeholder recommendations led to improvements in the software features (the ability to change age, store values, and return to the program later should patients wish to defer TKR), screens collecting the needed factors for the model variables, and visualization of the predicted outcomes.

#### KOMET system features

Based on stakeholder input, we designed the decision support to be able to be administered flexibly (prior to, during, and after a clinician visit) and to be able to be interpreted without direct clinician support or participation. Patient stakeholders wanted to be able to use the tool in advance of a visit in order to consider and discuss the results with family or friends. They also wanted to be able to store, retrieve, and print the reports over a period of time to track changes in the predictions as their condition changed. Clinicians also thought that it would be helpful for patients to arrive at the visit already having considered the information in order to focus the conversation. Clinicians believed that a number of different scenarios for using the information were likely, and these needed to be accommodated in KOMET. Not all patients would be interested in, or capable of, using the web-based tool, and some would need assistance completing the online questions and entering information. Clinicians also thought that there should be an alternative process for generating decision support using paper-based forms if the technology in the clinic could not support the online tool.

Both patient and clinician stakeholders were interested in the ability to modify model variables in a flexible way to view outcome predictions under different circumstances. For example, stakeholder input led to addition of a feature that allowed changing the patient’s age in the use of KOMET to understand if pain and function outcome predictions would change over time, such as if they waited for surgery until they retired, and if so, by how much would the predicted outcomes change.

#### Visualizations of predictions

**Fig. 1.**
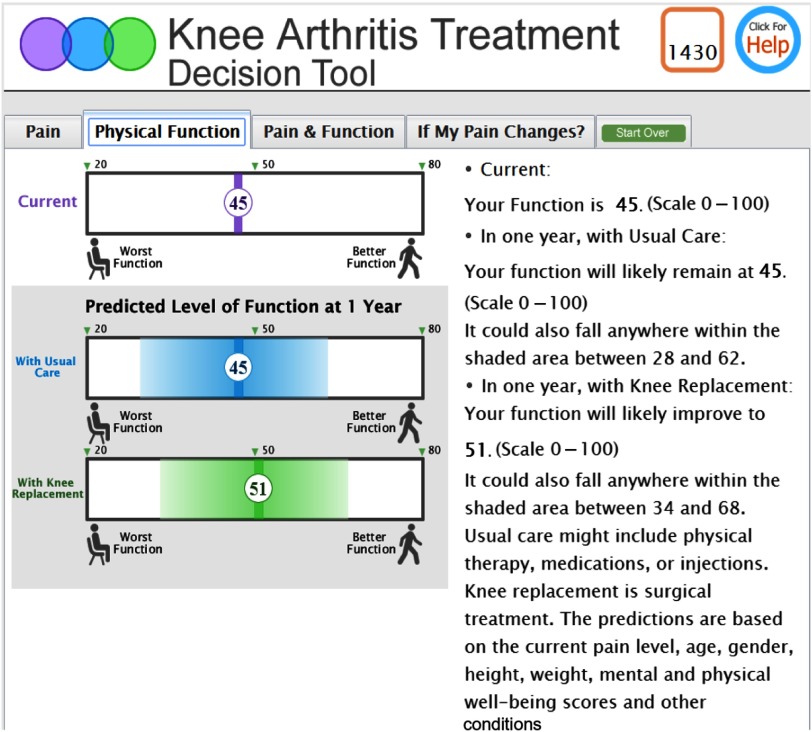
Physical function decision support graphic.

The iterative usability testing process addressed stakeholder feedback resulting in a final interface design. Pain and function decision support graphics display current scale scores for pain or function for a specific patient and that patient’s predicted outcomes in one year, with or without knee replacement. Fig. 1 is an example of a physical function graphic. The shaded area around the predicted score represents the range of possible values for the patient to convey the imprecision of the predictions.

We explored with stakeholders the depiction of outcome predictions using different types of scales and visualizations to determine which visualizations were best understood and most useful for decision-making. We also asked clinicians which visualizations would be easiest to explain during treatment decision-making. The final version addressed stakeholder comments by revising presentation features including the direction of scale values and the use of simple icons to represent better or worse function and more or less pain. Each of the outcome predictions included an area of uncertainty around the prediction. Some stakeholders had a difficult time understanding the depictions of uncertainty when represented as a graphic alone, so explicit wording was added to explicitly explain the range of predicted values for pain and function.

#### Results of usability testing

**Fig. 2.**
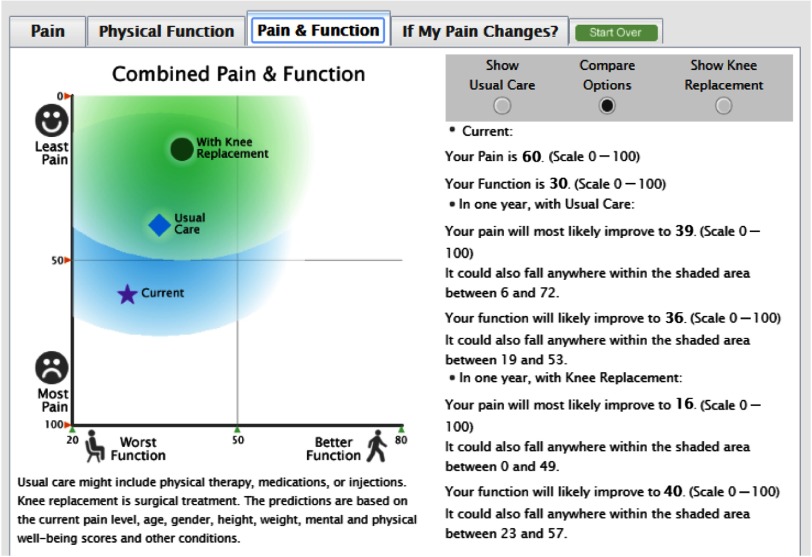
Knee Osteoarthritis Mathematical Equipoise Tool (KOMET) depiction of the combined predictions for pain and physical function.

Of the 10 patients who participated in usability testing in conjunction with the stakeholder clinicians, 80% (8/10) could describe the meaning of the patient-specific *current* pain and function scores and the *predicted future* pain and function outcomes as depicted by the bar graphs. Only 50% (5/10) of patients appeared to understand that the shaded area around the predictions represented the uncertainty (representing the 95% prediction interval for 1-year function and the 95% prediction interval for knee pain) around the predicted value. In reviewing the figure displaying combined pain and function probabilities and their related uncertainty circles as presented in Fig. 2, only 60% (6/10) were able to describe the meaning of the predicted outcomes, and 50% (5/10) could describe the meaning of the uncertainty circle around the prediction. The presentation of the predictions in the bar charts appeared to be easier to understand based on user comments and preferences.

Table 3 provides a sample of patients and clinician perspectives about the tool’s usefulness. Patients and clinician stakeholders were asked a series of questions to determine if they believed KOMET was useful for treatment decision support. Some patients believed the information provided additional information they had not considered previously. Others, not understanding or believing the predictions, did not find them helpful. Still others believed the decision support was consistent with their own beliefs about the expected outcomes of surgical or nonsurgical treatment. Clinicians were generally supportive of the decision support tool and any information that helped foster a clinician–patient conversation about the patient’s preferences, treatment goals, and establishing realistic expectations of treatment outcomes.

**Table 3.** Patient and clinician perception of the decision support tool’s usefulness for treatment decision-making

**Decision support tool provided information patients found useful:**
*It’s [the decision support results] telling me to get the surgery and to get rid of the pain and to return to my normal activity. It’s easier for me to make my mind up to do the surgery now* *I wouldn’t have a knee replacement. To me, I’m looking at these numbers and saying that they are not that bad. The pain is not that bad for me to do it*
**Decision support tool provided information that reinforced patients’ own beliefs about treatment outcomes:**
*It’s telling me what I think, that I’m not ready for knee replacement*
**Decision support tool did not provide information the patient found useful:**
*I actually think I would be better [than the results suggested with knee replacement]. I know that the doctor has to under promise. You can’t over promise*
**Decision support tool provided information the clinician believed supported clinician–patient decision-making:**
*The tool helped me to define her [the patient’s] expectations. She interpreted her costs and benefits. She wanted to be in the upper right quadrant [best function and least pain] if she were to have the knee replacement*. *Anything that helps patients think about what is important to them is helpful. It [the decision support results] helped her see that she would do better with surgery. It’s what we’ve been talking about for a while*.

The decision support visualizations were also intended to support decision-making about potential enrollment into a randomized controlled trial when the predicted outcomes suggested similar benefit between both treatments [13]. The graph in Fig. 2 depicts the combined predictions of pain and function in a single visualization. This figure was not easily understood by patient stakeholders without additional explanation. The stakeholder and user testing led the study team to conclude that the concept of mathematical equipoise and methods to depict it in context of predicted outcomes were not clear despite repeated redesigns of the user interface. Additional methods for depicting the concept need to be explored in future work.

## Discussion

Patient, clinician, and researcher stakeholders played an integral part in all aspects of this methodological project to develop mathematical models that predict patient-specific outcomes of treatment options for knee osteoarthritis and a decision support program. While stakeholders are increasingly involved in CER, the role of stakeholders in studies that involve using expert knowledge to develop innovative research *methods*, such as this study, is less apparent given the complexity of study activities. We addressed the fundamental considerations given stakeholder engagement in other types of research [22] with an emphasis on educating stakeholders on the project methods and promoting stakeholder beliefs about the importance, value, and impact of their involvement. Insights learned from stakeholder participation shaped the development of the predictive models, design and testing of KOMET, and plans for future refinements and implementation. We hope our experience informs other researchers on the role of stakeholders in methodological studies.

A challenge of engaging stakeholders in methodological research is to ensure that they have sufficient research training [23] and feel prepared to contribute their perspectives when decisions regarding the methodology are being made. To accomplish this, we provided patient and nonresearcher clinician stakeholders with an overview of the predictive modeling process using straightforward examples and sought their input on specific questions about the selection of the outcomes and model variables, and the usefulness of model output.

Our experience engaging stakeholders in developing the predictive models and designing and testing KOMET illustrates both benefits and challenges of stakeholder engagement in methodologic research. Stakeholders provided insight into the selection of outcomes and model variables thereby ensuring that the study team was including those outcomes most important to patients with severe knee osteoarthritis and not requiring a burdensome level of data collection. While patients agreed that pain and function outcomes were of central importance in their decision-making, they also raised other outcomes that could not be included given the study timeline and limitations of the available data. This reinforced the importance of clear communications about study limitations to ensure continued stakeholder engagement and realistic expectations.

KOMET’s design and planned use were informed by the stakeholders involved in its development and user testing. Their insight shaped KOMET content, our understanding of patients’ information needs in decision-making, informed the possible clinical or nonclinical settings for KOMET use, and KOMET’s ultimate form.

Most patient stakeholders found that viewing simple pain or function predicted outcome bar graphs provided a valuable perspective, but more complex outcome depictions did not achieve the intended goals. Combining the predicted outcomes for treatment options into a single visualization was not easily understood without additional detailed explanation. Our attempt to represent the imprecision of the risk estimates contributes to research literature about communicating uncertainty in decision support interventions [24]. We planned to use this illustration to support decision-making about potential enrollment into a randomized controlled trial when the predicted outcomes suggested similar benefit between treatments. The difficulties patients experienced in interpreting the combined prediction decision support prevented us from assessing if the information was useful in considering clinical trial enrollment. Overall, this limited stakeholder user testing provided positive proof of concept but further testing and revisions to the user interface are required.

There are a number of limitations to our approach. While we focused on those outcomes of importance to stakeholders, the selection of outcomes was limited by the availability of data for model development and the study timeframe. Patient stakeholders were interested in outcomes not represented in our modeling dataset and suggested that the predicted pain and function numerical scale composite outcomes available in the four datasets available to us be translated to important functional activities such as gardening or climbing stairs. This raises the issue of incorporating meaningful patient-centered outcomes into study design. This also illustrates the importance of reconsidering whether numerical outputs should be the only method for the decision support or if there are other options to better address patients for whom the numerical outputs are not as meaningful (problems with numeracy). Our project focus was limited to the benefits of each treatment decision despite the importance of considering both benefit and harm [25]. Finally, the timeframe of the project did not allow validation of the model or sufficient user testing and refinement to immediately implement KOMET in the clinical setting. Future work will test for model validity and incorporate harms as well as benefits.

Methodological research has the opportunity to benefit from stakeholder engagement by ensuring that the perspectives of those most impacted by the results are involved in study design, execution, and dissemination. While additional planning and investments in maintaining stakeholder knowledge and trust may be needed, they are offset by the valuable insights gained. Although there is a tradition of stakeholder engagement in clinical and community research, it has been less common in methodologic research. We think such input greatly benefited our project and encourage other efforts of this kind.
